# Does pre-existing L5-S1 degeneration affect outcomes after *isolated L4-5 fusion* for spondylolisthesis?

**DOI:** 10.1186/s13018-015-0186-8

**Published:** 2015-03-26

**Authors:** Kyung-Chul Choi, Hyeong-Ki Shim, Jin-Sung Kim, Sang-Ho Lee

**Affiliations:** Department of Neurosurgery, The Leon Wiltse Memorial Hospital, Anyang, Korea; Department of Neurosurgery, Prime Hospital, Busan, Korea; Department of Neurosurgery, Seoul St. Mary’s Hospital, College of Medicine, The Catholic University of Korea, 222 Banpo Daero Seocho-gu, 137-701 Seoul, Korea; Department of Neurosurgery, Wooridul Spine Hospital, Seoul, Korea

**Keywords:** Pre-existing degeneration, Anterior lumbar interbody fusion, Transforaminal lumbar interbody fusion, Adjacent segment disease, Spondylolisthesis, L5-S1

## Abstract

**Background:**

Concerns have been raised regarding residual symptoms of caudal segment (L5-S1) degeneration that may affect clinical outcomes or require additional surgery after isolated L4-5 fusion, especially if there is pre-existing L5-S1 degeneration. This study aimed to evaluate the L5-S1 segment after minimally invasive lumbar interbody fusion at the L4-5 segment, as well as the influence of pre-existing L5-S1 degeneration on radiologic and clinical outcomes.

**Methods:**

This retrospective study evaluated patients with isthmic spondylolisthesis and degenerative spondylolisthesis who underwent mini-open anterior lumbar interbody fusion with percutaneous pedicle screw fixation (PSF) or minimally invasive transforaminal interbody fusion with PSF at the L4-5 segment. The minimum follow-up period was 7 years, and radiographic evaluations were conducted via magnetic resonance imaging, computed tomography, and plain radiography at the 5-year follow-up. Clinical outcomes were assessed using the Visual Analog Score, Oswestry Disability Index, and surgical satisfaction rate. Patients were divided into two groups, those with and without pre-existing L5-S1 degeneration, and their final outcomes and incidence of radiographic and clinical adjacent segment disease (ASD) were compared.

**Results:**

Among 70 patients who underwent the procedures at our institution, 12 (17.1%) were lost to follow-up. Therefore, this study evaluated 58 patients, with a mean follow-up period of 9.4 ± 2.1 years. Among these patients, 22 patients had pre-existing L5-S1 degeneration, while 36 patients did not have pre-existing L5-S1 segmental degeneration. There were no significant differences in the clinical outcomes at the final follow-up when the two groups were compared. However, radiographic ASD at L5-S1 occurred in seven patients (12.1%), clinical ASD at L5-S1 occurred in three patients (5.2%), and one patient (1.7%) required surgery. In the group with pre-existing degeneration, L5-S1 degeneration was radiographically accelerated in four patients (18.2%) and clinical ASD developed in one patient (4.5%). In the group without pre-existing degeneration, L5-S1 degeneration was radiographically accelerated in three patients (8.3%) and clinical ASD developed in two patients (5.7%). There were no differences in the incidence of ASD when we compared the two groups.

**Conclusions:**

Pre-existing L5-S1 degeneration does not affect clinical and radiographical outcomes after isolated L4-5 fusion.

## Background

Adult spondylolisthesis predominantly presents as the isthmic type (abnormalities of the pars interarticularis) and the degenerative type (due to disc degeneration and facet arthropathy). Spondylolisthesis of the vertebral segments causes instability and neural compression, and the goal of surgical treatment is to achieve stabilization and decompression of the neural tissues. Although various surgical techniques have been used to treat spondylolisthesis, segmental fusion is a common and established treatment for spondylolisthesis. However, segmental fusion can affect the degenerative changes in adjacent segments, due to increased stress and motion. Thus, biomechanical stress on the disc and facet joints at the adjacent segments has been suggested to play a key role to the development of adjacent segment disease (ASD) after fusion [1,2]. Unfortunately, although ASD is considered a part of the aging process, it also requires surgery and can affect clinical outcomes. In addition to segmental fusion, many studies have reported that laminectomy, loss of lordosis, age, pre-existing degeneration at adjacent segment, and length of fusion are risk factors for ASD occurrence [3-6].

Interestingly, L4-5 spondylolisthesis is associated with caudal segment (L5-S1) degeneration, particularly among elderly patients, in whom L5-S1 degeneration is observed more frequently than any other type of degeneration. However, this also affects surgical decision-making when fusion is indicated for patients with L4-5 spondylolisthesis and concomitant L5-S1 degeneration, as the residual symptoms that are associated L5-S1 degeneration may affect clinical outcomes or require additional surgery after the isolated L4-L5 fusion. This concern is especially relevant if there is pre-existing L5-S1 degeneration. Therefore, we sought to evaluate the L5-S1 segment at 7 years after L4-5 minimally invasive lumbar interbody fusion and to determine if pre-existing L5-S1 degeneration influenced the radiologic and clinical outcomes.

## Methods

The protocol for this retrospective study was approved by Wooridul Spine Hospital institutional review board (WRDIRB-2013-04-007). At this institution, patients with isthmic spondylolisthesis and degenerative spondylolisthesis undergo mini-open anterior lumbar interbody fusion (mini-ALIF) with percutaneous pedicle screw fixation (PSF) or minimally invasive transforaminal interbody fusion (MIS-TLIF) with PSF for only the L4-5 segment.

Prior to the present study, two independent studies were conducted at our institution. The first study evaluated mini-ALIF for isthmic spondylolisthesis, and the 5-year and 10-year follow-up results have been reported previously [7,8]. The second study evaluated MIS-TLIF in the same institution, and the 5-year follow-up results have also been reported [9]. However, follow-up for both studies was continued, and the present study evaluated all cases of isolated L4-5 fusion for spondylolisthesis from the two previous studies. Among the 70 patients who originally underwent isolated L4-5 fusion via ALIF or MIS-TILF, 58 patients (82.9%) completed a minimum of 7 years of radiologic and clinical follow-up for the present study.

Radiographic evaluations were conducted via magnetic resonance imaging (MRI), computed tomography (CT), and plain radiography for all patients at the 5-year follow-up. Radiographs were assessed before surgery, after surgery, and at the final follow-up. Clinical and functional outcomes were assessed using the Visual Analog Score (VAS, 0–10 points) and the Oswestry Disability Index (ODI), respectively. The ODI is comprised of ten items, each of which contains six possible answers. Each item is scored from 0 to 5 points, and the sum of the scores is then presented as a percentage (0%–100%). In addition, the subjective surgical satisfaction rate (%) was assessed by asking the patient “How satisfied were you with this operation?”

The patients were divided into two groups: those with pre-existing L5-S1 degeneration and those without pre-existing L5-S1 degeneration. Pre-existing degeneration was defined as a disc degeneration grade of ≥4 [10], facet degeneration of ≥2 [11], foraminal stenosis, spinal canal stenosis, herniated nucleus pulposus, and instability. Instability was defined as a translation of 4 mm or 10° of angular motion, and foraminal stenosis was defined as fat obliteration on the T1-weighted sagittal image.

Radiographs, including the dynamic view, were analyzed by two blinded neurosurgeons (KCC and HKS) who were not involved in the surgeries. L5-S1 segmental angle was defined using the angle between the upper endplate of the L5 vertebral body and that of the S1 vertebral body. Pelvic tilt, pelvic incidence, and sacral slope were checked in lateral radiography. Bone fusion was assessed using CT reconstruction images and/or flexion-extension lateral radiographs. If there was <4° of movement in the fixed segment on the lateral view during flexion-extension, as well as continuity of the trabecular bony bridging across the disc space, the outcome was classified as “fusion.” If there was any movement observed on the lateral view during flexion-extension, or any discontinuity of the trabecular bony bridging, the outcome was classified as “pseudarthrosis.” An outcome of “probable fusion” was defined as lack of definitive continuity of the trabecular bony bridging, despite the absence of movement in the fixed segment during flexion-extension [12,13].

Disc height was calculated as the average of the anterior and posterior disc heights [14], and disc degeneration was graded via MRI based on the Pfirrmann grade, using the T2-weighted image at the midsagittal plane [10]. Facet degeneration was classified into four grades (0–3) using the grading system proposed by Weishaupt et al. [11] and was compared according to the width of the joint space, osteophyte formation, hypertrophy of the articular bone erosion, and the presence of subchondral cysts on the CT images. Radiographic ASD was diagnosed using the following criteria: (1) olisthesis (anterolisthesis or retrolisthesis) of >4 mm, (2) >10% loss in disc height, (3) >10° of angular motion between adjacent bodies on the flexion and extension radiographs, (4) osteophyte formation of >3 mm, (5) disc herniation or spinal stenosis on CT or MRI, (6) a change in disc degeneration of grade 2 or greater, (7) a change in facet arthropathy of grade 2 or greater, (7) scoliosis, or (8) compression fracture [15-21]. Clinical ASD referred to the development of new clinical symptoms that corresponded to radiographic ASD, such as a VAS score of ≥6 for the back or leg, an ODI score of >40%, or symptoms that required surgery [8].

We compared the surgical outcomes and incidence of radiographic and clinical ASD between the groups with and without pre-existing degeneration. All statistical analyses were performed using SPSS for Windows (version 14.0, SPSS, Inc., Chicago, IL). Intergroup differences were analyzed using Fisher’s exact test or the chi-square test, as appropriate, and results were considered statistically significant at a *p* value of <0.05.

## Results

**Figure 1:**
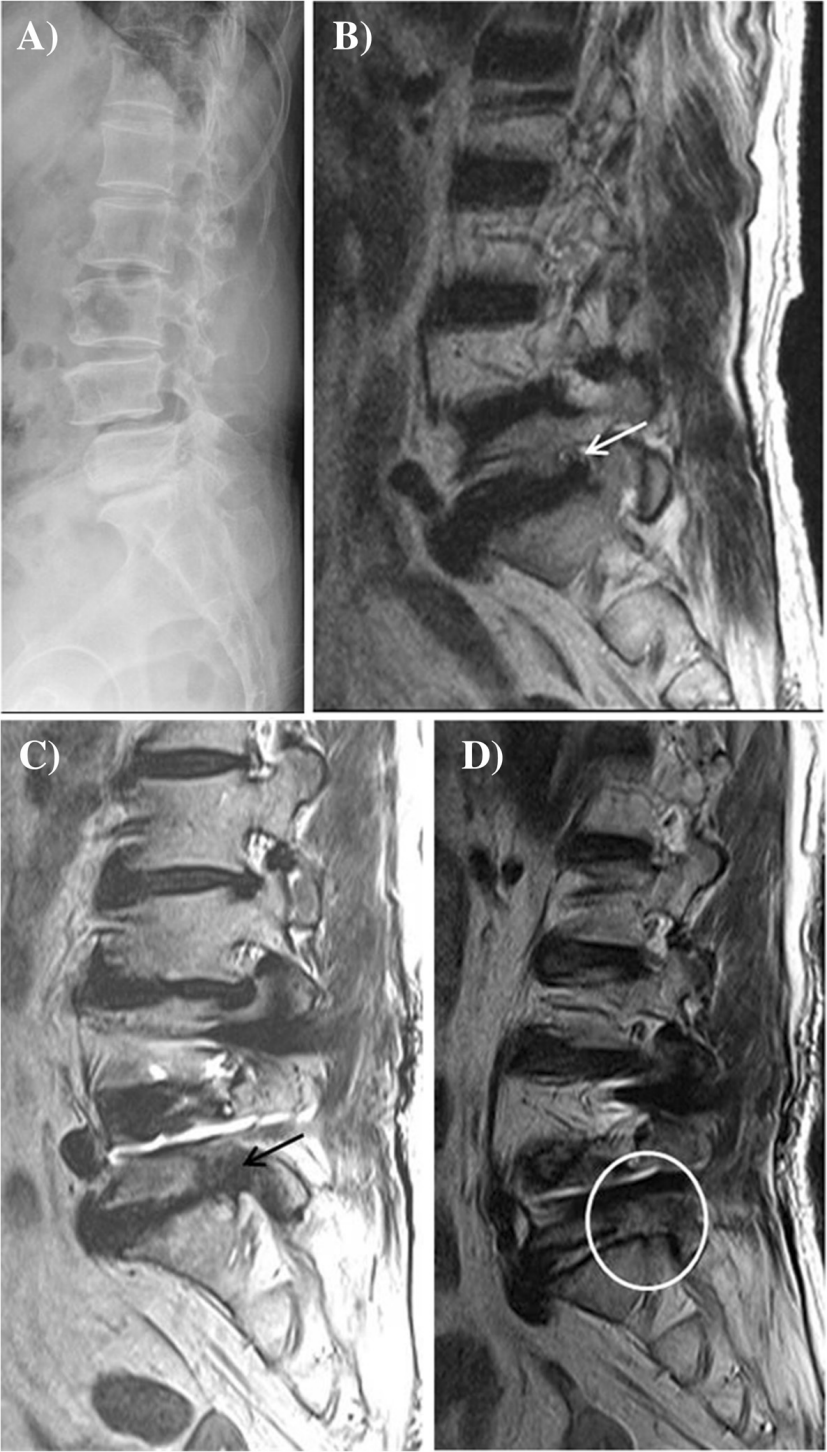
**Imaging of degenerative spondylolisthesis in a 65-year-old woman. (A)** Lateral radiography reveals degenerative spondylolisthesis at L4-5 and decreased disc height at the L5-S1 level. **(B)** T2-weighted right parasagittal magnetic resonance imaging (MRI) of the lumbar spine reveals spondylolisthesis at the L4-5 level and right foraminal stenosis (white arrow) of L5-S1. **(C)** MRI reveals aggravation of the foraminal stenosis (black arrow) of L5-S1 after anterior lumbar interbody fusion of L4-5 (10 years after surgery). **(D)** MRI reveals widening of the L5-S1 foramen (white circle) after decompression via the intermuscular approach at L5-S1.

Among the 70 patients who were originally treated in the two previous studies, 12 (17.1%) were lost to follow-up. Therefore, this study evaluated 58 patients, including 38 women and 20 men. The average age at surgery was 54.5 ± 8.3 years, and the mean follow-up period was 9.4 ± 2.1 years. Among the included patients, we observed 18 cases of degenerative spondylolisthesis and 40 cases of isthmic spondylolisthesis. MIS-TLIF was used in 30 cases, and mini-ALIF was used in 28 cases. The overall fusion rate, including both complete and probable fusions, was 96.6% (56/58). Radiographic ASD of L5-S1 occurred in seven patients (12.1%), clinical ASD of L5-S1 occurred in three patients (5.2%), and surgery was required for one patient (1.7%; Figure 1).

### Pre-existing L5-S1 degeneration versus no pre-existing L5-S1 degeneration

**Figure 2:**
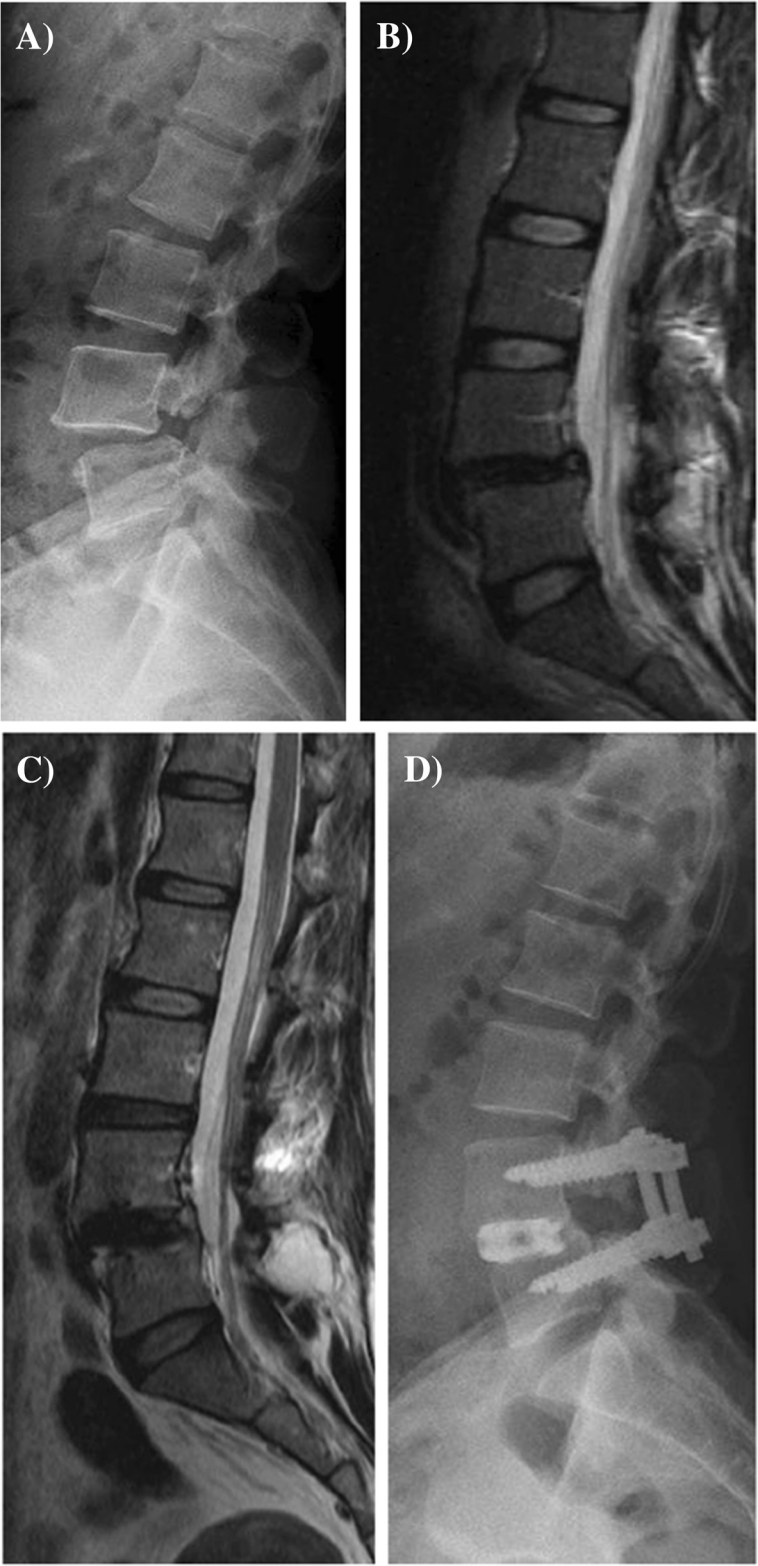
**Imaging of isthmic spondylolisthesis in a 49-year-old woman. (A)** Lateral radiography reveals L4-5 isthmic spondylolisthesis. **(B)** T2-weighted sagittal magnetic resonance imaging (MRI) reveals L4-5 spondylolisthesis without degeneration at the L5-S1 level. **(C)** Sagittal MRI reveals reduced slippage at the L4-5 level and no acceleration of degeneration at the L5-S1 level after minimally invasive transforaminal lumbar interbody fusion at L4-5 (5 years after surgery). **(D)** Seven years later, lateral radiography reveals complete interbody fusion of L4-5 and good maintenance of disc height at the L5-S1 segment.

Among the 58 included patients, 22 patients had pre-existing L5-S1 degeneration at surgery, while 36 patients did not have pre-existing L5-S1 degeneration (Table 1). In the pre-existing degeneration group, preoperative back and leg pain (VAS score) improved from 6.3 ± 2.9 and 7.0 ± 2.4 to 3.6 ± 2.7 and 2.8 ± 2.7 at the final follow-up, respectively, while the ODI score improved from 56% ± 15.3% to 23% ± 20.9%. In the group without pre-existing degeneration (Figure 2), preoperative back and leg pain improved from 6.3 ± 2.4 and 7.1 ± 1.9 to 3.1 ± 2.4 and 2.9 ± 2.7 at the final follow-up, respectively, while the ODI score improved from 55.2% ± 18% to 19.5% ± 13.3%. When we compared the two groups, no significant differences were observed for the postoperative ODI scores, back pain, leg pain, or satisfaction rates at the final follow-up (all, *p* > 0.05).

**Table 1:** **Comparison of patient characteristics according to pre-existing L5-S1 degeneration**

	**Pre-existing deg**	**Non-existing deg**	***p*** **value**
No	22	35	
M/F	6/13	16/23	*0.49*
Age	58.5 ± 6.7	54.1 ± 8.3	*0.11*
VAS pre back	6.3 ± 2.9	6.3 ± 2.4	*0.78*
VAS pre leg	7.0 ± 2.4	7.1 ± 1.9	*0.81*
ODI pre	56.0 ± 15.3	55.2 ± 18.0	*0.87*
VAS post back	3.6 ± 2.7	3.1 ± 2.4	*0.51*
VAS post leg	2.8 ± 2.7	2.9 ± 2.7	*0.94*
ODI post	23.0 ± 20.9	19.5 ± 13.3	*0.34*
Satisfaction rate	78.8 ± 16.7	79.2 ± 14.7	*0.98*
DH pre	9.5 ± 2.6	10.7 ± 2.6	*0.1*
DH post	9.3 ± 2.6	10.7 ± 2.7	*0.08*
PI	53.8 ± 9.7	55.3 ± 10.0	*0.59*
PT	20.9 ± 7.8	21.0 ± 8.4	*0.97*
SS	30.6 ± 9.0	33.9 ± 8.1	*0.18*
L5-S1 seg angle pre	16.4 ± 7.3	15.6 ± 6.6	*0.65*

M/F: male/female, VAS: Visual Analog Score, pre: preoperative, post: postoperative, ODI: Oswestry Disability Index, DH: disc herniation, PI: pelvic incidence, PT: pelvic tilt, SS: sacral slope, seg: segmental; *p* < 0.05 is statistically significant (data in italics).

L5-S1 degeneration was radiographically accelerated in four patients (18.2%) with pre-existing degeneration, and clinical ASD developed in one patient (4.5%) who underwent decompressive surgery for foraminal stenosis. Among the patients without pre-existing degeneration, L5-S1 degeneration was radiographically accelerated in three patients (8.3%), and clinical ASD developed in two patients (5.7%) who underwent open discectomy and nerve root block for newly developed radicular pain. However, there was no significant difference in the incidence of radiologic and clinical ASD between the two groups (Table 2).

**Table 2:** **The incidence of adjacent segment degeneration according to pre-existing degeneration of L5-S1**

	**Pre-existing deg**	**Non-existing deg**	***p*** **value**
Radiologic ASD Y	4 (18.2%)	3 (8.3%)	0.41
Radiologic ASD N	18 (81.8%)	33 (91.7%)	
Clinical ASD Y	1 (4.5%)	2 (5.7%)	1.00
Clinical ASD N	21 (95.5%)	34 (94.4%)	

ASD: adjacent segment disease, Y: yes, N: no.

## Discussion

Instrumented lumbar fusion is thought to be the gold standard treatment for lumbar instability. However, after instrumented fusion, mechanical changes can influence the adjacent segments of the facet joint and disc [15,19,22], and the resulting ASD can require further surgery and affect clinical outcomes [19]. Interestingly, ASD has been extensively studied [2,8,15,17,23], and the incidence of ASD has been reported to range from 5.2% to 100% [19]. In addition, many studies have demonstrated that ASD of the cranial segment occurs more frequently than that of the caudal segment. Furthermore, in a biomechanics study of the stresses that are associated with lumbar interbody fusion, the stress on the cranial adjacent segment was found to be larger than that on the caudal adjacent segment [1]. Although cranial segment degeneration or instability is reportedly caused by loss of lumbar lordosis, destruction of the superior interspinal ligament, and iatrogenic injury of the superior facet [3,24], only a few studies have evaluated caudal segment degeneration, especially at L5-S1 after isolated L4-5 fusion [18,25].

Decision-making is problematic for L4-5 spondylolisthesis with concomitant L5-S1 degeneration, as there is a concern that the L5-S1 degeneration could negatively affect clinical outcomes after isolated L4-5 fusion. In addition, two-level fusion at L4-5 and L5-S1 does not provide better outcomes than those obtained using single-level fusion [26], and the incidence of pseudarthrosis is higher in two-level fusion than that in single-level fusion [27]. Furthermore, L5-S1 fusion also affects sacroiliac joint degeneration [28]. Interestingly, the incidence rate of cranial ASD is higher for isolated lumbar fusion, compared to lumbosacral fusion, and resulting disability is more frequent [26], although preservation of L5-S1 motion may reduce buttock stiffness. In addition, a few studies have evaluated L5-S1 after isolated fusion or floating fusion, although these studies only reported plain radiography results with a relatively short radiologic follow-up period [26,29]. The authors conducted a 7-year follow-up radiologic evaluation using CT scan and MRI. Approximately 7 years after the L4-5 fusion, similar incidences of radiographic and clinical ASD in the L5-S1 segment were observed for patients with and without pre-existing L5-S1 degeneration (12.1% vs. 18.2% and 5.2% vs. 4.5%, respectively). In contrast, Park et al. have reported that pre-existing degeneration was correlated with L5-S1 ASD (10.7% of cases), that the fusion level was also correlated with L5-S1 degeneration, and that L5-S1 degeneration negatively affected clinical outcomes [29].

Several authors have suggested that older patients with L5-S1 degeneration do not exhibit compensatory movement at the L5-S1 level, although L5-S1 angular displacement increased among middle-aged patients after L4-5 posterior lumbar interbody fusion [30]. In addition, Ghiselli et al. studied L5-S1 survivorship after isolated fusion, with radiologic and clinical follow-up periods of 3.9 years and 7.3 years, respectively [25]. According to their report, the resulting L5-S1 survivorship was 90%, and L5-S1 degeneration was only identified on the radiographic findings, which did not affect the patients’ clinical symptoms. However, given that most symptomatic ASD is not usually observed during short-term follow-up, especially in segments without pre-existing degeneration, long-term follow-up is essential to evaluating outcomes at the L5-S1 level after isolated fusion [19].

Preoperative L5-S1 disc space narrowing does not affect clinical outcomes after L4-5 posterior lumbar interbody fusion [18]. In addition, among cases with advanced L5-S1 degeneration, lumbosacral fusion did not achieve better clinical results than lumbar floating fusion, which indicated that that L5-S1 disc degeneration did not affect the final clinical outcomes [26]. Furthermore, in thoracolumbar fusion for spinal deformity, fusion at the L5 level achieves good outcomes, although L5-S1 degeneration can result in sagittal imbalance [6]. The subsequent degeneration in the L5-S1 segment occurred in 69% of cases (per the radiologic aspects), and surgery was required in 23% of cases after thoracolumbar fusion was stopped at the L5 level. Finally, Kim et al. have reported that the incidence of pseudarthrosis was higher when the procedure was extended to the sacrum, compared to when it was stopped at L5 [31,32]. In this context, the strong iliolumbar ligament supports the L5 vertebra and ilium and also stabilizes the L5-S1 segment in the pelvis, and the L5-S1 facet joints rarely are violated using screws or muscle dissection. In contrast to the previous studies, all patients in the present study underwent mini-ALIF and MIS-TLIF with percutaneous pedicle screws, which did not violate the posterior back muscles and supraspinous ligament, which may have had a positive effect on the extended survivorship of the L5-S1 level. Similarly, Penta et al. have also suggested that ALIF does not accelerate degeneration of the adjacent intervertebral discs [20].

There is one major limitation in this study. If pre-existing L5-S1 degeneration was advanced or was associated with symptoms, we excluded patients who underwent two-level fusion or additional decompression at the L5-S1 level. Therefore, future studies are needed to compare lumbosacral fusion to lumbar floating fusion sparing L5-S1 segment.

## Conclusions

Pre-existing L5-S1 degeneration does not affect clinical and radiographical outcomes after isolated L4-5 fusion. Therefore, it may not be necessary to include L5-S1 fusion in cases of L4-5 spondylolisthesis with concomitant L5-S1 degeneration if the preoperative symptoms are not attributed to the L5-S1 level.

## Abbreviations

mini-ALIF: Mini-open anterior lumbar interbody fusion
MIS-TLIF: Minimally invasive transforaminal interbody fusion
PSF: Percutaneous pedicle screw fixation
ASD: Adjacent segment disease
VAS: Visual Analog Score
ODI: Oswestry disability index
MRI: Magnetic resonance imaging
CT: Computed tomography
